# Cross‐Night Modulation of Change Detection ERPs to Foreign Speech Sound Features During N2 Sleep

**DOI:** 10.1111/ejn.70670

**Published:** 2026-09-16

**Authors:** Qin Li, Jari L. O. Kurkela, Jarmo A. Hämäläinen, Xueqiao Li, Piia Astikainen

**Affiliations:** ^1^ Department of Psychology University of Jyväskylä Jyväskylä Finland; ^2^ Faculty of Social Sciences/Psychology Tampere University Tampere Finland

**Keywords:** auditory oddball, event‐related potentials, sleep study

## Abstract

The sleeping brain can discriminate speech sounds, but evidence for cross‐night changes in neural responses to foreign speech in adults remains limited. Here, Finnish‐speaking adults naïve to tonal languages were exposed during Stage N2 of non‐rapid eye movement (NREM) sleep to small and large deviations in tone (pitch contour) embedded in vowel /a/ across four consecutive nights, while electroencephalography was recorded. Event‐related potentials (ERPs) P2 and P450 revealed robust change detection responses to tone deviations during sleep, and importantly, P450 amplitude decreased from night 1 to night 4. No evidence of perceptual learning was found in waking change detection ERPs or in behavioural performance measured with the same stimuli before and after the four‐night sleep exposure. In conclusion, the sleeping brain remained sensitive to deviations in vowel tone, and the amplitude reduction of the late ERP deflection suggests a change in auditory processing across nights of exposure. However, in the absence of transfer effects to wakeful measures, these findings do not provide evidence for phonetic learning‐related plasticity, and the extent to which the effects are speech‐specific remains unclear. These findings provide new insight into the dynamics of auditory processing during sleep and contribute to broader neuroscientific understanding of sensory processing in altered states of consciousness.

## Graphical abstract

During N2 sleep, Finnish‐speaking adults naïve to tonal languages showed auditory change detection responses to tonal deviations, as indexed by P2 and P450. Across four consecutive exposure nights, P450 amplitude was reduced on Night 4 compared to Night 1, whereas P2 amplitude and latencies remained unchanged. The absence of corresponding changes in waking ERPs or behavioural performance suggests cross‐night attenuation of auditory responses during sleep rather than clear evidence of perceptual learning.

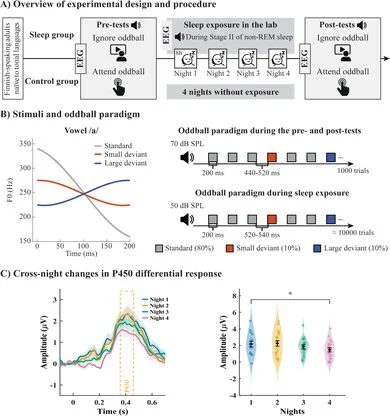

**Abbreviations**
ANOVA: Analysis of variance
EEG: Electroencephalography
ERP: Event‐related potential
M: Mean
MMN: Mismatch negativity
NREM: Non‐rapid eye movement
REM: Rapid eye movement
SD: Standard deviation
SE: Standard error
SPL: Sound pressure level
SWS: Slow‐wave sleep

## 1 Introduction

Decades of research demonstrate that the sleeping adult brain can acquire new information (e.g., Weinberg 1966; Schmidig et al. 2024; for a review, see Ataei et al. 2023). Associative learning during sleep has been demonstrated across modalities, including the learning of tone–odour (Arzi et al. 2012) and word–meaning associations (Koroma et al. 2022). Even if neural speech perception mechanisms are partly functional in sleep (Atienza et al. 2001), relatively little is known about perceptual learning of foreign speech sounds during sleep in adults. This is because most studies have been conducted in infants or by exposing adult participants to simple non‐speech stimuli, typically within a single experimental session (Ataei et al. 2023), with multi‐night exposure paradigms remaining largely unexplored. Here, we show cross‐night modulation of late auditory event‐related potentials (ERPs) during N2 sleep in adults exposed to foreign speech sound features, i.e., tonal changes embedded in a repeatedly presented vowel /a/.

Phonetic learning constitutes a fundamental step in language acquisition, as successful mastery of a new language requires sensitivity to fine‐grained acoustic cues that distinguish phonemes (Kuhl 2004). During infancy, overnight exposure to novel vowel contrasts improves neural discrimination specifically for these sounds (Cheour et al. 2002). However, in adults, neural plasticity is reduced relative to infancy, and extensive native language experience stabilises phonetic categories (Kuhl 2004; Kuhl 2008), potentially limiting the acquisition of novel phonetic features and associated experience‐dependent neural changes (Pallier et al. 1997; Flege et al. 1999). In addition, several studies have failed to show effects of passive exposure on auditory change detection in adults even in awake state (Näätänen et al. 1993; Sheehan et al. 2005; Elmer et al. 2017). However, our previous studies have demonstrated that passive exposure to a foreign tonal feature for four consecutive days in an awake state can modulate neural indices of change detection within a few hours (Kurkela et al. 2019; Li et al. 2025), suggesting that some degree of neural plasticity is preserved even in adulthood for phonetic learning in the absence of explicit attention. However, it remains unclear whether repeated exposure to foreign speech sounds during sleep in adults can modulate their neural representation across multiple nights.

Here, we applied an auditory oddball paradigm during sleep exposure, consistent with previous studies in sleeping humans investigating change detection in speech (Blume et al. 2017) and non‐speech sounds (Atienza et al. 2001; Côté 2002; Colrain and Campbell 2007). In this stimulus condition, infrequent ‘deviant’ sounds embedded within a sequence of frequent ‘standard’ sounds typically elicit the mismatch negativity (MMN), an ERP response associated with automatic change detection (Näätänen et al. 1978; Näätänen et al. 2007). MMN is typically investigated in an ignore condition where participant is attending to other stimulus modality (Näätänen et al. 2007), but the response is also elicited in altered states of consciousness, such as coma (Fischer et al. 1999, 2000) and rapid eye movement (REM) sleep (Atienza et al. 1997, 2000; Atienza and Cantero 2001). However, during NREM sleep, MMN‐like responses are less consistently observed than during wakefulness (Colrain and Campbell 2007). This has been interpreted within a broader framework of sensory gating during sleep, reflecting a balance between sleep protection and environmental monitoring (Coenen 2024). Notably, the canonical waking P300, reflecting preattentive shift toward deviant sounds (Escera et al. 1998; Friedman et al. 2001; Polich 2007), is not reliably observed during stable NREM sleep (Pratt et al. 1999; Côté 2002; Colrain and Campbell 2007). Instead, in the Ndr literature, components such as P2, N350, P450 and N550 are often described as part of a broader auditory response sequence (Campbell and Colrain 2002; Bastien et al. 2002). Studies vary in which components are reported, with some covering most of the sequence and others focusing on specific ERPs such as N350/N550 (Côté et al. 1999; Colrain et al. 2000; Czisch et al. 2009).

P2 is an auditory ERP which is not specific to sleep or to oddball paradigms. Its precise functional significance during sleep remains uncertain (Crowley and Colrain 2004; Campbell and Colrain 2002). N350 appears closely linked to sleep onset, NREM physiology and vertex sharp wave activity, and may reflect an early evaluation or gating of external stimulation during light sleep (Colrain et al. 2000; Bastien et al. 2002). P450 is observed in sleep oddball or salient‐stimulus paradigms and can be sensitive to stimulus probability or salience, but it has been suggested that it is not a direct equivalent of the waking P3 (Hull and Harsh 2001). N550 is closely tied to sleep physiology and is widely regarded as the dominant negative component of the evoked K‐complex, serving as an index of NREM‐specific cortical responses to external stimulation (Côté et al. 1999; Cash et al. 2009; Czisch et al. 2009). None of these components are specific to change detection, because similar responses can also be elicited by standard tones, respiratory stimuli, personally meaningful stimuli or other non‐oddball auditory stimulation (Colrain et al. 2000; Perrin et al. 1999; Côté et al. 1999).

Here, we exposed adult native speakers of Finnish with no prior experience of tonal languages to an isolated vowel /a/ with occasional Mandarin tone‐like changes. The aim was to investigate the effects of multi‐night exposure on sleep ERPs and, in the case of robust learning effects, potential transfer to change detection performance in the awake state. The stimuli were presented during N2 sleep across four nights, while electroencephalography (EEG) was recorded. We selected to use exposure during N2 sleep because change detection responses have been reliably demonstrated in NREM sleep, and because N2 is a clearly identifiable stage in polysomnographic recordings that typically constitutes a substantial proportion of total sleep time (Colrain and Campbell 2007; Berry et al. 2012).

In wakefulness, before and after sleep exposure, ERP and behavioural change detection were measured for the same oddball sound series as in sleep exposure. To capture both automatic and attention‐dependent aspects of auditory change processing, waking ERPs were recorded in ignore and attend oddball conditions. In the ignore condition, participants passively listened to tone sequences while watching silent movies. In the attend condition, participants monitored the stimuli and responded to target tones with button presses. A control group, which participated only in the pre‐ and post‐recordings, was included to control effects of repeated testing unrelated to sleep exposure.

We hypothesised that rare changes (deviants) in tone, compared with the frequent standard tone in the vowel /a/, would elicit differential neural responses during N2 sleep, reflecting auditory change detection. In addition to amplitude, ERP latency provides information about the timing of neural stimulus processing and may be sensitive to experience‐ or learning‐related changes in auditory processing (Picton et al. 2000; Duncan et al. 2009). Therefore, the amplitude and possibly latency of differential responses were expected to change across nights in the sleep exposure group, reflecting modulations in the neural processing of novel speech sound features.

It is unclear whether possible sleep exposure effect could transfer to wakefulness recordings, either at the neural or behavioural level. Robust transfer of newly encoded information to waking neural or behavioural measures is relatively rare in literature and is typically observed only in paradigms involving prior wakeful encoding or explicit memory reactivation (Ataei et al. 2023; Paller and Voss 2004). While our design allowed us to assess such transfer, we adopted a more conservative expectation for waking measures, as the primary focus was on sleep‐related neural responses and their modulation across nights. Therefore, we expected robust change detection responses (MMN and N2b) and attention‐shifting responses (P3a and P3b) in awake ERP recordings but made no predictions about exposure‐related effects (comparison between experimental and control groups).

## 2 Method

### 2.1 Participants

An a priori power analysis was conducted using G*Power (α = 0.05, power = 0.80). Because the central hypothesis concerned systematic modulation of ERP responses across consecutive nights of sleep exposure, the power analysis was aligned with a within‐subject repeated‐measures design (Night; four levels). In the absence of pilot data and directly comparable multi‐night sleep ERP studies from which to derive an empirical effect size, we adopted a convention‐based medium effect size (Cohen's *f* = 0.25; Cohen 1988) as a planning assumption. The analysis indicated that a minimum of 24 participants would be required to detect a medium‐sized within‐subject effect.

Participants were recruited via university mailing lists and noticeboard postings at the University of Jyväskylä. Eligibility criteria required participants to be 18**–**30 years old, right‐handed, with normal hearing verified by audiometry (≤ 20 dB hearing level at 250–1000 Hz), and normal or corrected‐to‐normal vision. Individuals with a history of neurological or psychiatric illness, including sleep‐related disorders, or with prior training or long‐term exposure to tonal languages were excluded. Short‐term exposure (no more than 2 weeks) during travel to countries where tonal languages are spoken was accepted.

In total, 41 native Finnish speakers volunteered to take part in the experiment. They were assigned to either a sleep group (*n* = 20; mean age = 23.0 years, *SD* = 1.93) or a control group (*n* = 21; mean age = 24.1 years, *SD* = 3.6). However, group allocation was not randomised. Data collection for the two groups occurred during partially overlapping time periods. The control group corresponds to the non‐sleep exposure sample reported in Kurkela et al. (2019), with different research questions and analyses in the present study. All participants gave written informed consent before the study. The protocol was approved by the University of Jyväskylä Ethics Committee, and the study was conducted in accordance with the Declaration of Helsinki.

### 2.2 Stimulus Presentation

The same auditory stimuli were presented during sleep exposure and in the awake pre‐ and post‐tests. In an oddball paradigm, a standard stimulus occurred on 80% of trials, while two types of deviant stimuli (large and small deviant) each appeared on 10% of trials. Stimuli were presented in pseudorandom order, with at least two standard stimuli between successive deviant stimuli.

Tone variation—an unfamiliar phonetic feature for the participants—served as the target feature during sleep exposure. Speech materials were first recorded by a female Mandarin native speaker and then edited in SoundForge and Praat. A pitch‐tier transfer procedure allowed the manipulation of fundamental‐frequency (F0) contours while keeping all other acoustic properties constant, yielding two endpoint tokens that differed only in pitch contour (rising vs. falling). Linear interpolation between these endpoints produced an 11‐step tonal continuum. The duration of each token was 200 ms. Stimulus preparation is described in detail in Xi et al. (2010); in the present study, we used only the vowel portion.

**FIGURE 1:**
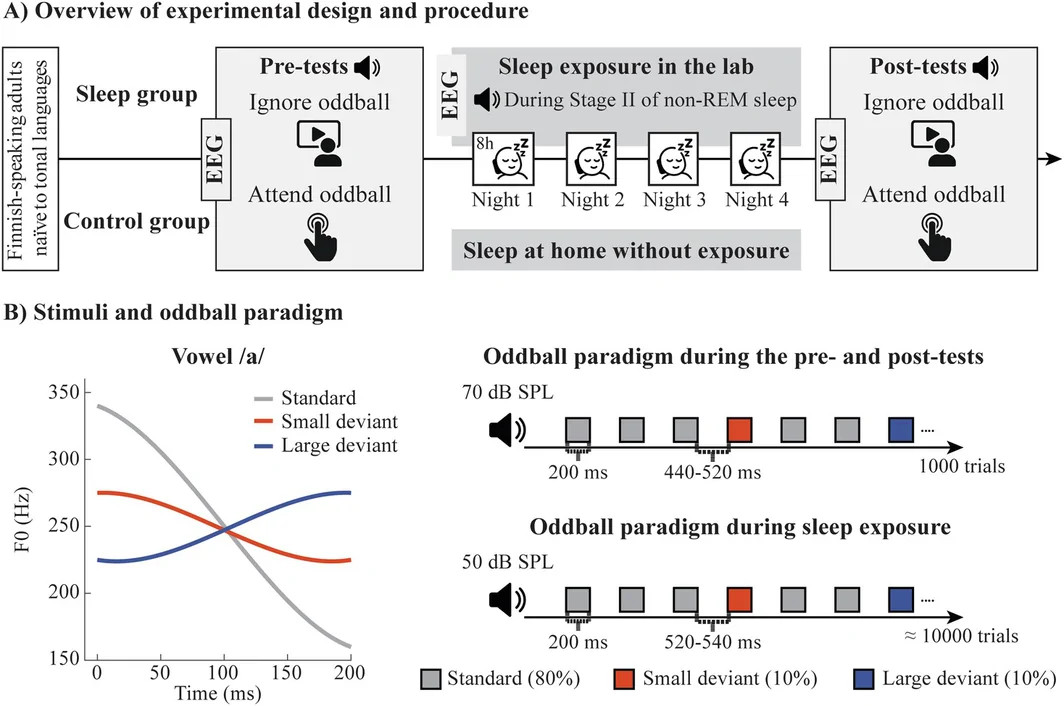
Overview of the experimental design and auditory oddball paradigms. (A) Participants in the Sleep group underwent four consecutive nights of sleep exposure in the laboratory. During each exposure night (Nights 1–4), auditory stimuli were presented during N2 sleep while EEG was continuously recorded. The Control group slept at home without exposure. Both groups completed pre‐ and post‐test sessions during wakefulness, in which they either ignored the auditory oddball sequences while focusing on a silent movie or actively attended to the sequences by pressing a button in response to deviant stimuli. (B) Stimuli and oddball paradigm. The vowel /a/ with different tones (F0 contours) were applied as Standard, Small deviant and Large deviant stimulus.

The falling tone served as the standard stimulus, while the slightly falling and rising tones (Figure 1) served as the Small and Large deviants, respectively. Including two deviant types reduced the risk of ceiling or floor effects that could arise from presenting only a highly salient or overly subtle tone contrast. The interstimulus interval (offset‐to‐onset) varied randomly between 440 and 520 ms in the pre‐ and post‐test sessions, and between 520 and 540 ms during sleep exposure. Stimuli were delivered using *E‐Prime* 1.2 software (Psychology Software Tools Inc., Sharpsburg, Pennsylvania, United States).

Pre‐ and post‐test procedures are described in detail in Kurkela et al. (2019). In these sessions, 1000 stimuli were presented at an intensity of 70 dB SPL through a loudspeaker positioned approximately 50 cm above the participant's head.

During sleep exposure, substantially more stimuli (~10,000 per night) were presented, with the exact number depending on N2 sleep duration for each participant (reported below). Sound intensity gradually increased from 0 dB to 50 dB over the first 3 min and remained at 50 dB thereafter to avoid disrupting sleep. Sounds were delivered through a loudspeaker positioned approximately 1 m from the participant.

### 2.3 Procedure

The pre‐tests were conducted on a day immediately preceding the first sleep exposure, and the post‐tests were conducted in the morning after the last sleep exposure (Figure 1). During the pre‐ and post‐tests, participants were seated in a comfortable chair in a well‐lit room, with a video connection to the experimenter. Two experimental conditions were employed: ignore and attend. In the ignore condition, participants were instructed to ignore the sounds and watch a silent movie. In the attend condition, they were asked to press a response button as quickly as possible upon detecting a deviant stimulus, with no movie presented.

During the exposure phase, participants in the sleep group spent four consecutive nights in the laboratory, sleeping individually in a separate bedroom. Each night, participants remained in the bedroom for a maximum of approximately 8 h (typically from 23:00 to 07:00). Polysomnographic signals were monitored continuously by two trained researchers who identified N2 sleep based on the presence of characteristic sleep spindles and K‐complexes (Tatum et al. 2007). Auditory stimulation commenced when N2 sleep was reliably detected. Sound exposure was paused whenever the participant transitioned out of N2 and resumed once N2 sleep was re‐established.

### 2.4 EEG and Polysomnography Recordings

During the pre‐test and post‐test sessions, raw EEG data were acquired using a 128‐channel Hydrogel Geodesic Sensor Net (Electrical Geodesics Inc., Eugene, Oregon, United States) with Ag–AgCl electrodes. Signals were sampled at 500 Hz and online filtered between 0.1 and 200 Hz.

During sleep exposure, polysomnography was recorded using the NeurOne system (Mega Electronics Ltd., Kuopio, Finland). EEG was recorded from electrodes C3, C4, FPz and M2 positioned according to the international 10–20 system, with M1 serving as the reference and a forehead electrode as ground. Electrodes were attached using medical‐grade adhesive. This electrode configuration allowed sleep staging and assessment of auditory responses within the practical constraints of overnight recording, but it did not permit detailed evaluation of the classical frontal topography often emphasised in studies of K‐complexes and related slow‐wave activity. Electro‐oculogram signals were recorded from electrodes placed at the outer canthi, and chin electromyogram was obtained using a bipolar mentalis–submentalis configuration. All electrophysiological signals were sampled at 250 Hz and filtered online between 0.1 and 200 Hz.

Sleep stages were analysed offline using YASA (Vallat and Walker 2021), an automated sleep staging toolbox based on machine‐learning classifiers trained on expert‐scored polysomnographic data, to verify that auditory stimulation occurred predominantly during N2 sleep, as intended. YASA has been shown to achieve high agreement with manual sleep staging, with particularly strong performance for N2 sleep classification (Vallat and Walker 2021). The analysis confirmed that auditory exposure was tightly constrained to the targeted sleep stage: For each participant, 99.1%–100% of all stimulation epochs occurred during N2 sleep.

Across four nights, the average duration of auditory exposure during N2 sleep was 91 min on Night 1 (*SD* = 56.15, range = 0–176 min), 137 min on Night 2 (*SD* = 16.77, range = 119–180 min), 127 min on Night 3 (*SD* = 36.23, range = 2–157 min) and 132 min on Night 4 (*SD* = 24.97, range = 75–176 min).

On average, sleep consisted of 6.6% N1, 49.9% N2, 19.6% SWS and 24.2% REM sleep. Sleep architecture followed the canonical nocturnal pattern, with a higher proportion of SWS sleep occurring in the first part of the night and a progressive increase in REM sleep toward morning.

Although sleep studies often include an adaptation night to reduce the first‐night effect, that is, changes in sleep architecture and quality associated with an unfamiliar laboratory environment (Agnew et al. 1966), Night 1 was retained here because it marked the onset of auditory exposure rather than a separate adaptation night, allowing inclusion of all available data and assessment of changes from the onset of stimulation.

### 2.5 Preprocessing and Statistical Analysis of Pre‐ and Post‐Tests EEG Data

Because no robust effects of sleep exposure were observed in pre‐ versus post‐recordings relative to the control group, the corresponding methods and results are reported in the Supplementary Materials.

### 2.6 Preprocessing and Statistical Analysis of Sleep EEG Data

Data preprocessing and analysis were performed using EEGLAB (Delorme and Makeig 2004) and FieldTrip (Maris and Oostenveld 2007), supplemented by custom MATLAB scripts (The MathWorks, Natick, Massachusetts, United States; R2019a). The raw EEG signals were first band‐pass filtered offline between 1 and 30 Hz and notch‐filtered at 50 Hz to suppress line noise. Continuous data were segmented into epochs extending from −100 to 700 ms relative to stimulus onset, followed by baseline correction using the mean voltage of the pre‐stimulus interval (−100 to 0 ms). Epochs were sorted into four categories: standards immediately preceding small deviants, small deviants, standards immediately preceding large deviants and large deviants. Trials with voltage fluctuations exceeding ±120 μV were rejected from further analysis. Participants were required to have at least 100 accepted trials in each epoch category on a given night to ensure an adequate signal‐to‐noise ratio. When this criterion was not met for a specific epoch category on a particular night, data from that night were treated as missing rather than excluding the participant from the analysis. Accordingly, the final sample with usable ERP data comprised 15, 20, 19 and 20 participants for Nights 1–4, respectively.

ERP analyses were conducted on signals averaged across C3, C4 and FPz electrodes. The present study only focused on the P2 and P450, as the N350 and N550 deflections were not sufficiently well‐defined in the grand‐average waveforms to determine consistent and reliable time windows across conditions; these ERPs were not included in the statistical analyses.

The analysis windows were selected based on previous sleep ERP literature reporting P2 and P450 (Winter et al. 1995; Bastien et al. 2002; Colrain and Campbell 2007), and were finally confirmed by grand‐average differential responses (deviant − standard) averaged across participants, the four nights, and deviant types to minimise circularity (Kriegeskorte et al. 2009). For each component, the peak latency was defined as the time point of the maximum positive deflection in the collapsed grand‐average difference wave within the literature‐guided search range. Based on these peak latencies, symmetrical analysis windows were defined around each component (±35 ms for P2 and ±50 ms for P450), resulting in windows of 221–291 ms and 358–458 ms after stimulus onset, respectively.

Mean amplitude of differential response (deviant‐standard) was extracted for all participants for responses on each night and differential response type (Small and Large change) within these windows. Latency was estimated using the 50% area latency method (Kiesel et al. 2008; Luck 2014), which is less sensitive to local peak fluctuations than peak latency. For each deviant‐minus‐standard difference wave, latency was defined as the time point at which 50% of the total positive area within the selected time window of the positive component was reached.

### 2.7 Statistics for Sleep Exposure Data

To verify that auditory tone changes elicited reliable ERP responses during the initial exposure night, Night 1 deviant‐minus‐standard mean amplitudes were tested against zero using one‐sample t‐tests separately for P2 and P450 to confirm the presence of an initial differential ERP response.

To evaluate cross‐night changes in ERP responses relative to Night 1, we employed linear mixed‐effects models as implemented in MATLAB R2019a (MathWorks, Natick, Massachusetts, United States) using the *fitlme* function and the Statistics and Machine Learning Toolbox. Separate models were applied for the amplitude and latency of the differential response for P2 and P450. *Night*, *Deviant Type* and the *Night × Deviant Type* interaction were included as fixed effects, while *Participant* was included as a random effect with a random intercept.

To assess whether variability in exposure duration influenced the Night‐related ERP effects, two complementary analyses were conducted. First, likelihood‐ratio‐based model comparisons tested whether adding exposure duration as a covariate improved the fit of the ERP models. Its inclusion did not significantly improve model fit for any of the four measures (all *p*s > 0.263). Therefore, the more parsimonious model without exposure duration was retained for the primary analyses. Second, we examined whether exposure duration differed across nights after applying the trial‐count exclusion criterion described in Section 2.6. Because usable ERP data were not available for all participants on every night, exposure duration was analysed using a linear mixed‐effects model, with Night included as a fixed effect and participant included as a random intercept. Exposure duration did not differ significantly across nights, *F*(3, 70) = 1.79, *p* = 0.157.

Model convergence was confirmed for each fitted model. Residuals were inspected visually using residual histograms and quantile–quantile plots to assess approximate normality, and residual‐versus‐fitted plots were examined to evaluate homoscedasticity. No major deviations were observed that warranted further model modification.

The significance of the Night × Deviant type and Night main effect were assessed from the mixed‐model analysis of variance (ANOVA) using Satterthwaite approximation. If the Night × Deviant type or Night main effect reached significance, planned pairwise comparisons were performed between Night 2 and Night 1, Night 3 and Night 1, and Night 4 and Night 1. These contrasts were selected a priori because Night 1 represented the initial exposure baseline, and the primary research question was whether sleep ERP responses changed after repeated exposure to the Mandarin tone contrasts. Comparing each later night with Night 1 therefore directly tested whether the neural response deviated from the initial response at successive stages of exposure, including the cumulative change by Night 4. This planned contrast structure also avoided an exhaustive set of all pairwise comparisons and reduced the number of statistical tests. Bonferroni correction was applied to account for multiple night pairwise comparisons. Statistical significance was defined as *p* < 0.05 (two‐tailed). Effect sizes were reported as partial eta squared $\left(\eta_{p}^{2}\right)$ for omnibus fixed effects, correlation coefficients $\left(r\right)$ for planned pairwise comparisons, and Cohen's d for one‐sample tests against zero.

## 3 Results

**FIGURE 2:**
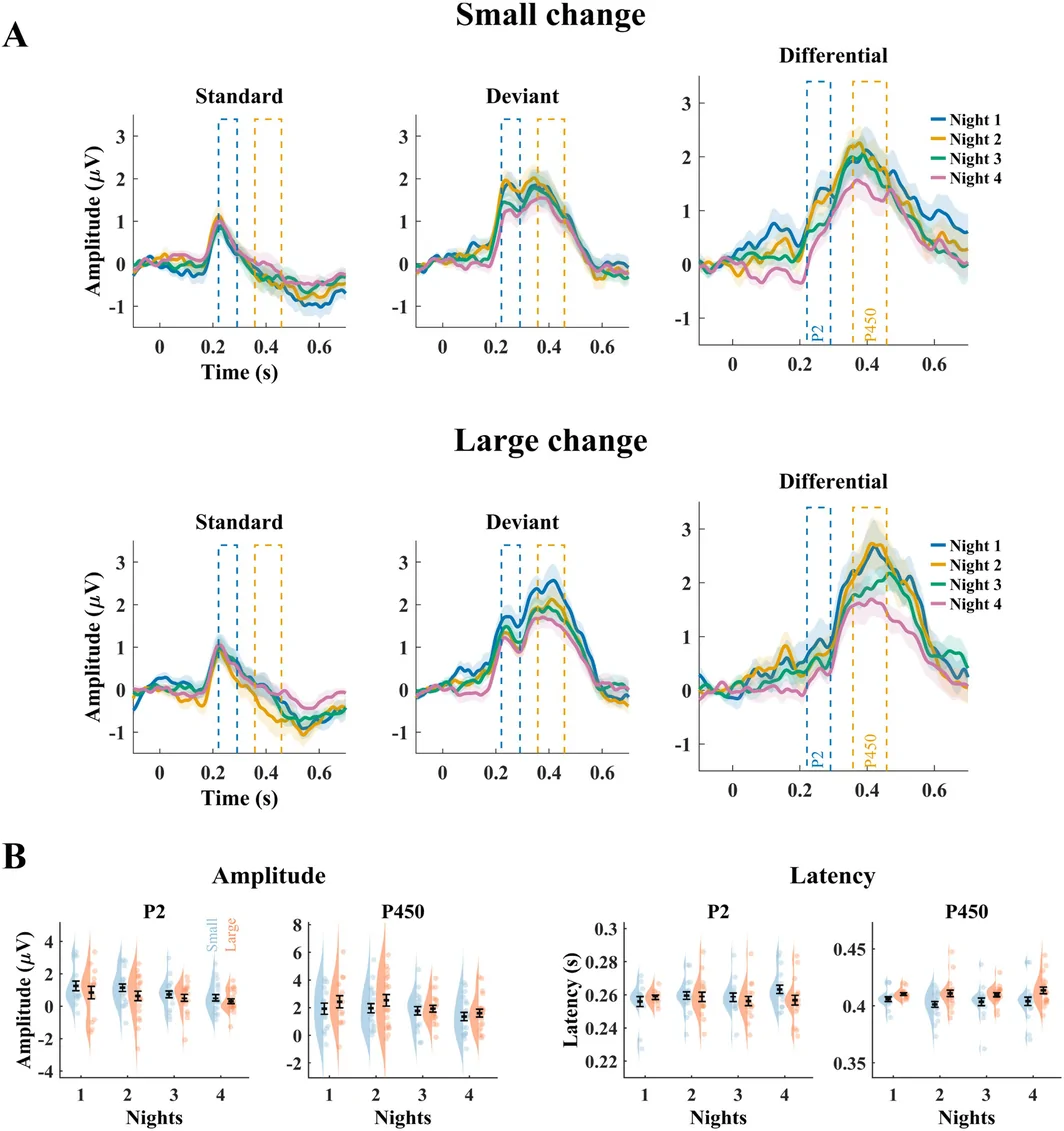
ERP waveforms and the corresponding amplitude and latency measures across the four exposure nights for the Small and Large change conditions. (A) Grand‐averaged ERPs for standard, deviant and differential responses (deviant—standard, averaged over C3, C4, FPz) separately for Small and Large change. The time windows used for P2 and P450 amplitude analyses (mean values at 221–291 ms and 358–458 ms post‐stimulus, respectively) are indicated. Shaded areas represent ±1 *SE* of the mean. Note that differential responses were used in the amplitude and latency analyses. (B) Mean ± *SE* of differential amplitudes and latencies. ‘Small’ refers to the Small Change condition, ‘Large’ refers to the Large Change condition. Each dot represents one participant's value for a given night; black symbols indicate the group mean ± *SE*, and the shaded half‐violin illustrates the data distribution.

**FIGURE 3:**
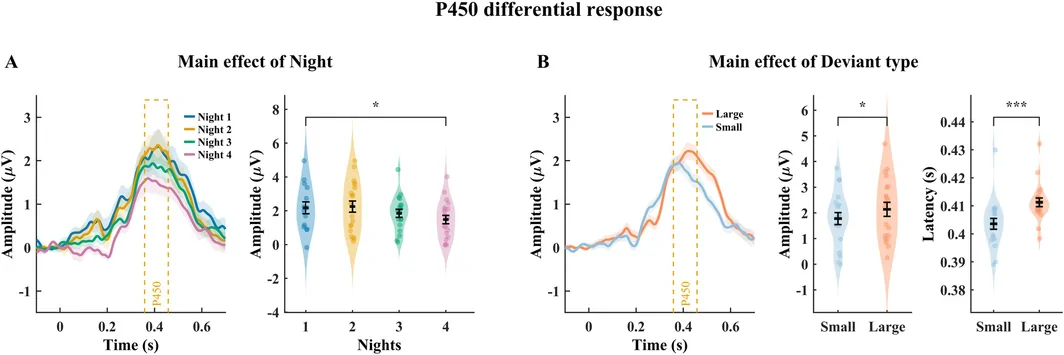
Main effects of Night and Deviant Type for P450 differential responses. (A) Grand‐average differential waveforms (deviant minus standard), averaged across Small and Large change conditions, for Nights 1–4, together with the corresponding distributions of mean P450 amplitude. (B) Grand‐average differential waveforms, averaged across the four nights, for Small and Large change conditions, together with the corresponding distributions of P450 mean amplitude and fractional‐area latency. Waveforms represent the average across FPz, C3 and C4, and shaded areas indicate ±1 *SE*. The orange dashed boxes indicate the P450 measurement window (358–458 ms). In the violin plots, dots represent individual‐participant values, and black markers and error bars indicate the mean ±1 *SE*. Asterisks indicate significant results for main effect of Deviant type and post hoc tests for main effect of Night (significant difference between Nights 1 and 4): *p* < 0.05; **p < 0.001.

Figure 2 provides a descriptive overview of the ERP waveforms and the corresponding amplitude and 50% fractional‐area latency measures across the four exposure nights for the Small and Large change conditions. Descriptive statistics for all ERP measures are reported in Table 1. Figure 3 presents the significant P450 effects identified by the mixed‐effects analyses. Complete ANOVA results for the linear mixed‐effects models are presented in Table 2.

**TABLE 1:** Mean and standard error (SE) of P2 and P450 amplitudes (μV) and latencies (ms) by night and deviant type.

ERP	Measure	Deviant type	Night	Mean	SE
P2	Amplitude	Small	1	1.26	0.30
			2	1.14	0.23
			3	0.73	0.21
			4	0.52	0.20
		Large	1	0.84	0.38
			2	0.63	0.29
			3	0.52	0.21
			4	0.31	0.15
	Latency	Small	1	255.97	3.00
			2	259.54	2.24
			3	258.51	2.53
			4	263.30	2.53
		Large	1	258.51	1.31
			2	258.74	2.82
			3	256.23	2.79
			4	256.80	2.94
P450	Amplitude	Small	1	1.92	0.40
			2	1.96	0.33
			3	1.77	0.31
			4	1.36	0.30
		Large	1	2.42	0.44
			2	2.54	0.42
			3	1.92	0.24
			4	1.61	0.29
	Latency	Small	1	405.90	2.17
			2	401.25	2.53
			3	403.54	3.33
			4	404.07	3.74
		Large	1	410.43	1.22
			2	410.93	2.82
			3	409.78	1.85
			4	413.62	2.87

**TABLE 2:** ANOVA results from linear mixed‐effects models for ERP amplitudes (μV) and latencies (ms).

ERP measure	Effect	F	df₁	df₂	p	$\eta_{p}^{2}$
P2 amplitude	Night	2.50	3.00	140.00	0.062	0.051
	Deviant Type	3.71	1.00	140.00	0.056	0.026
	Night × Deviant Type	0.19	3.00	140.00	0.905	0.004
P2 latency	Night	0.53	3.00	122.00	0.663	0.013
	Deviant Type	0.86	1.00	122.00	0.357	0.007
	Night × Deviant Type	0.95	3.00	122.00	0.420	0.023
P450 amplitude	Night	4.00	3.00	121.78	0.009*	0.090
	Deviant Type	4.16	1.00	120.76	0.044*	0.033
	Night × Deviant Type	0.32	3.00	120.76	0.811	0.008
P450 latency	Night	0.59	3.00	117.57	0.624	0.015
	Deviant Type	17.21	1.00	115.15	< 0.001**	0.130
	Night × Deviant Type	0.57	3.00	115.45	0.636	0.015

*Note:* *F* tests are from linear mixed‐effects models including Night, Deviant Type and the Night × Deviant Type interaction as fixed effects, with participant included as a random intercept. *df₁* = numerator degrees of freedom; *df₂* = denominator degrees of freedom; $\eta_{p}^{2}$ = partial eta squared.

* *p* < 0.05.

** *p* < 0.001.

### 3.1 Differential Responses on Night 1

We first investigated whether tone changes elicited reliable differential ERP responses on the initial exposure night, which served as the reference condition for the cross‐night analyses. The one‐sample *t*‐tests showed significant positive differential response amplitudes for both P2, *M* = 1.05 μV, *SD* = 1.31, *t*(29) = 4.38, *p* < 0.001, Cohen's SD = 0.80, and P450, *M* = 2.17 μV, *SD* = 1.62, *t*(29) = 7.34, *p* < 0.001, Cohen's SD = 1.34. Thus, reliable differential responses were present for both ERP responses on the first exposure night.

### 3.2 Cross‐Night Effects

The Night × Deviant Type interaction was not significant for any amplitude or latency measure, all *p*s ≥ 0.420.

The mixed‐effects analysis showed a significant main effect of Night for P450 differential amplitude, *F*(3, 121.78) = 4.00, *p* = 0.009, $\eta_{p}^{2}$ = 0.090. Bonferroni‐adjusted planned comparisons showed that the differential response for P450 amplitude was lower on Night 4 than on Night 1, with an estimated marginal‐mean contrast of −0.73 μV (Night 4 − Night 1), *SE* = 0.27 μV, *t*(122.58) = −2.72, adjusted *p* = 0.022, whereas amplitude values for Night 2 and Night 3 did not differ from Night 1, adjusted *p*s ≥ 0.979 (Figure 3A).

The main effect of Night was not significant for P2 differential amplitude or for the 50% fractional‐area latency of either P2 or P450, all *p*s ≥ 0.062.

### 3.3 Effects of Deviant Type

The mixed‐effects analysis showed a significant main effect of Deviant Type for both P450 amplitude, *F*(1, 120.76) = 4.16, *p* = 0.044, $\eta_{p}^{2}$ = 0.033 and latency, *F*(1, 115.15) = 17.21, *p* < 0.001, $\eta_{p}^{2}$ = 0.130. Large change condition elicited greater differential amplitudes than Small change condition, with an estimated marginal‐mean contrast of 0.37 μV (Large − Small), *SE* = 0.18 μV (Figure 3B). Large change condition also elicited later differential fractional ‐area latencies than Small change condition, with an estimated marginal‐mean contrast of 8 ms (Large − Small), *SE* = 2 ms. No significant effects of Deviant Type were observed for P2 amplitude or latency, both *ps* ≥ 0.056.

## 4 Discussion

The present study examined whether exposure to foreign speech sounds during nocturnal sleep modulates neural responses underlying change detection and whether such effects transfer to the awake state. Robust P2 and P450 responses were identified from sleep recordings. Both P2 and P450 responses showed robust deviant‐standard amplitude difference already on the initial exposure night. Importantly, the P450 amplitude exhibited significant cross‐night modulation. The P450 differential amplitude showed a significant reduction by Night 4. However, multi‐night sleep exposure did not result in amplitude modulation in awake recordings, dissociating these findings from perceptual learning and plasticity.

### 4.1 Change Detection in Foreign Tonal Features During N2

Robust auditory change detection responses were observed during N2 sleep, expressed in the P2 and P450 differential responses between deviant and standard sounds. The differential response to deviant tonal features was already present on Night 1, suggesting reliable neural sensitivity to tone changes. The finding is consistent with previous ERP studies using non‐speech sounds showing that auditory changes modulate both early and later positive ERP responses during drowsiness and N2 sleep (Winter et al. 1995). The P2 is a relatively early auditory evoked response that is sensitive to acoustic stimulus characteristics, although its precise functional significance in sleep remains incompletely understood (Campbell and Colrain 2002; Crowley and Colrain 2004). Together, the P2 and P450 differential responses suggest that auditory changes continued to be differentiated during N2 sleep, consistent with previous evidence that P210 and P430 responses during N2 sleep are sensitive to the magnitude of auditory deviance (Winter et al. 1995).

### 4.2 Cross‐Night Changes in Sleep ERPs

This study is the first to show that the adult brain can detect changes in foreign phonetic contrasts during nocturnal sleep, and that sleep‐related auditory responses exhibit measurable cross‐night neural modulations. Specifically, P450 differential response amplitude was lower on the fourth than on the initial exposure night, with no significant changes observed on the intervening nights.

This pattern in P450 amplitude suggests that changes in neural activity emerge with a delay, in contrast to the ERP changes we previously observed during passive exposure to similar tonal stimuli in the awake state, where significant changes were evident already after the first exposure session (Li et al. 2025). This slower time course may reflect the reduced level of external sensory processing during sleep compared with wakefulness.

In the present study, the neural changes were neither accompanied by reliable transfer to measures obtained during wakefulness nor by behavioural improvement, providing no evidence for stable perceptual learning of foreign speech sounds during sleep. Similarly, in the passive exposure study, we found no behavioural changes specific to the exposure group (see Kurkela et al. 2019); the control participants in that study were drawn from the same participant pool as in the present study. The effects of passive exposure during wakefulness (Kurkela et al. 2019; Li et al. 2025) and sleep exposure in the present study both differ from our previous study using a change detection paradigm in active training, where we observed robust behavioural improvement in tone perception and related neural plasticity already after the first day of training (Jiang et al. 2026). Instead, the present findings indicate differences in neural responses across repeated exposure sessions, with effects emerging only after multiple nights. Whether such overnight changes could eventually lead to stable perceptual learning with continued nocturnal exposure remains an open question.

Previous waking auditory studies have shown that P2 amplitude can be sensitive to repeated sound exposure and auditory experience. For example, P2 amplitude has been reported to increase after repeated exposure or training, sometimes even when behavioural improvement is not clearly demonstrated (Sheehan et al. 2005; Tremblay et al. 2010). Evidence from magnetoencephalography recordings has also shown increased P2m responses after passive listening and active training, possibly reflecting enhanced auditory object representation (Ross et al. 2013). In the present study, however, no significant cross‐night modulation was observed for P2. Therefore, the present findings do not provide evidence that repeated exposure to foreign speech sounds during N2 sleep modulated early P2‐range auditory processing. This absence of a significant P2 effect should be interpreted cautiously, however, because non‐significant results do not constitute evidence for the absence of an effect (Altman and Bland 1995). Moreover, previous P2 findings discussed above were obtained during wakefulness (Sheehan et al. 2005; Tremblay et al. 2010; Ross et al. 2013), whereas the present study examined auditory processing during N2 sleep. Thus, the lack of a reliable P2 effect may reflect differences in vigilance state, stimulation or statistical sensitivity, rather than a direct contradiction of previous waking P2 findings. Direct evidence that repeated auditory exposure modulates the canonical P2 response during N2 sleep remains non‐existent.

The interpretation of the cross‐night ERP modulation should also be considered in relation to variability in exposure duration. Although exposure duration varied across participants and nights, additional analyses did not indicate that this variability accounted for the observed ERP changes. Thus, we interpret the findings more cautiously as cross‐night ERP modulation during repeated sleep exposure, rather than as evidence for exposure‐driven neural modulation or a dose–response effect. This distinction is important because the present data do not provide evidence that exposure duration was associated with the magnitude of ERP modulation. However, the requirement for a minimum number of accepted trials resulted in fewer participants contributing usable ERP data on the first night compared with subsequent nights, which may have reduced the sensitivity of analyses involving exposure duration and ERP modulation. Future studies with sufficient trial numbers and more systematic control of exposure‐related factors are needed to determine whether sleep‐related auditory ERP modulation depends on the amount of exposure.

### 4.3 Effects of Deviant Type on Sleep ERPs

Although no interaction was found between Night and Deviant Type, indicating that the modulation across nights did not differ between the deviant types, a significant main effect of Deviant Type was observed. Overall, large changes elicited greater P450 differential amplitudes than small changes, consistent with a previous study showing enhanced P430 responses to larger deviations in non‐speech sounds during sleep (Winter et al. 1995). Together, these findings suggest that late sleep‐related auditory processing is sensitive to the magnitude of auditory change.

Here we also found latency difference for P450 differential responses for Small and Large change conditions, suggesting a modestly later temporal distribution for the Large change. Fractional‐area latency reflects the distribution of activity within a predefined window and can be influenced by waveform duration, morphology, and overlap with adjacent activity (Kiesel et al. 2008). Therefore, without possibility for source localisation, it is difficult to interpret this finding. We speculate that this difference can reflect a more sustained response related to stimulus evaluation for Large change instead of slower initial change detection.

### 4.4 Limitation and Future Direction

A main limitation of the present study is the absence of a control condition during sleep. Because no alternative auditory exposure was included, it remains unclear whether the observed reduction in P450 amplitude was specifically related to repeated exposure to foreign speech stimuli, whether it reflects more general speech or auditory processing (e.g., also present for native‐language or non‐speech sounds), or whether it arises from broader changes in brain activity unrelated to stimulus‐specific processing. To disentangle these possibilities, future studies should include control conditions that systematically vary the type of auditory input during sleep.

Another limitation of the present study concerns statistical power. Although the study was planned on the basis of a medium within‐subject effect, the effective sample for the main cross‐night ERP analysis was smaller than the planned sample size. This reduced sample size limits the precision of the estimates and the ability to detect small‐to‐moderate effects.

Finally, because the control group was drawn from a non‐randomised sample, it cannot be assumed to be fully equivalent to the sleep group. Accordingly, comparisons involving the control group should be interpreted cautiously, and the control condition is best viewed as a contextual comparison rather than a definitive randomised control.

## 5 Conclusions

In conclusion, the present study shows that foreign tonal contrasts can elicit auditory change detection responses during N2 sleep, as reflected by P2 and P450 differential responses. Cross‐night changes under repeated exposure were observed for the P450 amplitude, whereas effects in other measures were not robust. Overall, the findings do not provide evidence for broad sleep‐related learning effects of foreign speech sounds but rather indicate a limited and ERP deflection‐specific pattern of neural changes across repeated exposure sessions.

## Author Contributions

**Qin Li:** conceptualization, funding acquisition, writing – original draft, methodology, visualization, writing – review and editing, formal analysis. **Jari L. O. Kurkela:** conceptualization, investigation, methodology, writing – review and editing. **Jarmo A. Hämäläinen:** conceptualization, investigation, methodology, writing – review and editing. **Xueqiao Li:** methodology, visualization, writing – review and editing. **Piia Astikainen:** conceptualization, funding acquisition, investigation, methodology, writing – review and editing, project administration, supervision, resources.

## Funding

The study was supported by Research Council of Finland (grant number 351009 for Piia Astikainen) and China Scholarship Council (personal grant for Qin Li). The authors thank the students of Department of Psychology, University of Jyväskylä, for their help in data collection.

## Ethics Statement

The ethical committee of the University of Jyväskylä approved the research protocol. The experiment was undertaken in accordance with the Declaration of Helsinki.

## Conflicts of Interest

The authors declare no conflicts of interest.

## Supporting information


**Table S1:** Results of mixed‐effects repeated measures examining the interaction between Group (Control, Sleep) and Session (Pre, Post) for ignore condition, separately for each Deviant type (Small, Large) and each dependent variable. Reported statistics include *F*‐values with corresponding degrees of freedom (df₁, df_2_), *q* = FDR‐corrected p values (Benjamini–Hochberg), partial η^2^ = effect size (small ≈ 0.01, medium ≈ 0.06, large ≈ 0.14). For each measure, parameter estimates (β) and standard errors (SE) are also presented.
**Table S2:** Results of mixed‐effects repeated measures examining the interaction between Group (Control, Sleep) and Session (Pre, Post) under the attend condition, separately for each Deviant type (Small, Large) and each dependent variable. Reported statistics include F‐values with corresponding degrees of freedom (df₁, df2), q = FDR‐corrected p values (Benjamini–Hochberg), partial η2 = effect size (small ≈ 0.01, medium ≈ 0.06, large ≈ 0.14). For each measure, parameter estimates (β) and standard errors (SE) are also presented.
**Table S3:** Results of mixed‐effects repeated measures examining the interaction between Group (Control, Sleep) and Session (Pre, Post) for behavioural data, separately for each Deviant type (Small, Large) and each dependent variable. Reported statistics include F‐values with corresponding degrees of freedom (df₁, df2), q = FDR‐corrected p values (Benjamini–Hochberg), partial η2 = effect size (small ≈ 0.01, medium ≈ 0.06, large ≈ 0.14). For each measure, parameter estimates (β) and standard errors (SE) are also presented.
**Table S4:** Mean and standard error for the amplitude (μV) and latency (ms) of the ERPs in the ignore condition separately for Deviant type, Group and Session.
**Table S5:** Mean and standard deviation for the amplitude (μV) and latency (ms) of the ERPs in the attend condition separately for Deviant type, Group and Session.
**Table S6:** Mean and standard error for the behavioural accuracy (ACC, %) and reaction time (RT, ms) separately for the Deviant type, Group and Session.
**Figure S1:** Grand‐average differential responses and scalp topographies for the Control and Sleep groups under Ignore and Attend conditions, and for small and large changes.

## Data Availability

The raw data are not publicly available due to legal restrictions. The data that support the findings of this study are available upon reasonable request from Piia Astikainen (piia.astikainen@jyu.fi). The codes used in the analysis are published in GitHub ‐ AsophiliaChance/Sleep_exposure.
