# Supplementary material for: Cross‐Night Modulation of Change Detection ERPs to Foreign Speech Sound Features During N2 Sleep
Source: Eur J Neurosci. 2026 Sep 16;64(6):e70670. doi: 10.1111/ejn.70670 (PMC13579495; doi:10.1111/ejn.70670)
Supplement: Supplementary file 1 [file EJN-64-0-s001.pdf]

Supplementary materials

Cross-Night Modulation of Change Detection ERPs to Foreign Speech Sound Features During N2 Sleep

Qin Li, Jari L.O. Kurkela, Jarmo A. Hämäläinen, Xueqiao Li, and Piia Astikainen

Pre- and post-tests analyses

Behavioural data analyses

For behavioural data, a response was scored as a hit if it occurred within the response window extending from the offset of the target deviant to the onset of the second subsequent standard stimulus. This criterion allowed responses during the first post-deviant standard stimulus to be included as hits. Reaction times for hit responses were calculated from deviant onset.

Preprocessing of EEG data

Data preprocessing and analyses were performed using EEGLAB (Delorme & Makeig, 2004) and FieldTrip (Oostenveld et al., 2011), supplemented by custom MATLAB scripts (The MathWorks, Natick, MA, USA; R2019a). The analyses code is available on GitHub: https://github.com/AsophiliaChance/Sleep_exposure.

Raw EEG data were downsampled to 250 Hz, low-pass filtered at 30 Hz, and notch-filtered at 50 Hz to remove line noise. Muscle-related artefacts were rejected using spectrum-based thresholding with a 10-dB threshold within the 35–128 Hz frequency range. Independent component analyses (ICA) were then applied to the continuous data to identify and remove components associated with ocular activity. The cleaned data were segmented into epochs spanning from −100 ms to 600 ms relative to stimulus onset and baseline-corrected by subtracting the mean voltage of the −100 to 0 ms pre-stimulus interval. Epochs in which the voltage at any channel and time point exceeding ±200 µV were excluded from further analyses. EEG signals were re-referenced to the common average reference and averaged separately for each participant and stimulus type. The difference waves were computed by subtracting the standard-stimulus average from the deviant-stimulus average.

Analyses in the attend condition focused on the N2b and P3b components, measured at central (5, 6, 11, 12, 29, 30, 36, 37, 87, 104, 105, 111) and parietal electrodes (47, 52, 59, 60, 61, 62, 72, 78, 85, 91, 92, 98), respectively, using Electrical Geodesics Inc. (EGI; Eugene, OR, USA)

128-channel HydroCel Geodesic Sensor Net (GSN 128, 1.0). In the ignore condition, the mismatch negativity (MMN) and P3a components were examined at frontal electrodes (3, 4, 11, 16, 19, 20, 23, 24, 28, 117, 118, 124). The spatial layout of these electrode selections is illustrated in Figure S1. To define the amplitude analysis windows, a grand-average waveform collapsed across all participants, two sessions (pre- and post-test) and two conditions (Small and Large change) was first computed for the ignore and attend conditions separately to avoid circularity or "double dipping" in selection of the analyses windows. Prominent negative or positive deflections were then identified, and the corresponding peaks were used to guide the selection of fixed-length time windows. For each component, a symmetrical analysis window was defined around the corresponding peak: ±30 ms for MMN and N2b, and ±35 ms for P3a and P3b. This resulted in time windows of 182–242 ms for MMN, 210–270 ms for N2b, 257–327 ms for P3a, and 369–439 ms for P3b. Mean amplitudes were then extracted from the difference waves, calculated as deviant minus standard, within these intervals. Component latency was defined as the time point of the maximum negative deflection for negative components (MMN and N2b) or the maximum positive deflection for positive components (P3a and P3b) within the same window as Kurkela et al. (2019). Deviant stimulus responses, rather than differential responses, were used in the latency analyses because their shaper peak morphology allowed more reliable detection of response peaks.

Statistical analyses of behavioural and EEG data

Repeated-measures data were analysed using linear mixed-effects models (LMMs). Models were fitted separately for each deviant condition (Small change, Large change) and each dependent variable. For the attend condition, separate models were conducted for N2b amplitude, N2b latency, P3b amplitude, P3b latency, behavioural accuracy (ACC), and reaction times (RT). For the ignore condition, separate models were conducted for MMN amplitude, MMN latency, P3a amplitude, and P3a latency. The models included Group (Control vs. Sleep), Session (Pre vs. Post), and their interaction as fixed effects, and random intercepts for participants to account for repeated measurements (Model: Value ~ Group × Session + (1|Subject)). The significance of fixed effects was evaluated using Type III ANOVA with Satterthwaite-approximated degrees of freedom. For each fixed effect, effect sizes were

quantified using partial η^2, which represents the proportion of variance uniquely explained by the effect after accounting for other model terms. Following conventional benchmarks, values of approximately .01, .06, and .14 were interpreted as small, medium, and large effects, respectively. To control for multiple comparisons across components and measures, p values were adjusted using the Benjamini–Hochberg false-discovery-rate (FDR) procedure, and statistical conclusions were based on the resulting q values.

All statistical analyses were implemented in MATLAB R2019a (The MathWorks, Natick, MA, USA) using the *fitlme* function and associated routines from the Statistics and Machine Learning Toolbox.

Beyond the analyses of traditional ERP components, we conducted exploratory analyses guided by visual inspection of the grand-average waveforms. For the ignore condition, the same statistical approach was applied separately to mean amplitudes extracted from consecutive 50-ms bins within the 350–500 ms interval.

Results

No significant Group × Session interactions, which would indicate sleep-exposure-related improvement, were observed (Table S1-3). In both the ignore and attend conditions, ERP amplitudes and latencies (MMN, P3a, N2b, P3b) showed no reliable enhancement from pre- to post-test. Similarly, reaction times and accuracy showed no evidence of group-specific or session-related improvement. Together, these results indicate that passive exposure to tones during nocturnal sleep did not lead to measurable enhancement in auditory discrimination, attentional processing, or behavioural performance during wakefulness. Means and standard errors (SE) for ERP and behavioural measures by task condition, deviant condition, group, and session are presented in Tables S4-S6.

Also, exploratory bin-wise analyses of the ignore-condition waveforms did not reveal any significant Group × Session interactions within the 350–500 ms interval. This was the case for

all consecutive 50-ms time bins, indicating no reliable group difference in pre-to-post changes in this later time range.

Table S1. Results of mixed-effects repeated measures examining the interaction between Group (Control, Sleep) and Session (Pre, Post) for ignore condition, separately for each Deviant type (Small, Large) and each dependent variable. Reported statistics include F-values with corresponding degrees of freedom (df_1, df_2), q = FDR-corrected p values (Benjamini–Hochberg), partial η^2 = effect size (small ≈ .01, medium ≈ .06, large ≈ .14). For each measure, parameter estimates (β) and standard errors (SE) are also presented.

ERP	Measure	Deviant	Estimate	SE	$F_{(1,72)}$	q	partial η^2
MMN	Amplitude	Small	-0.15	0.39	0.14	.941	0.002
		Large	-0.31	0.31	0.98	.870	0.013
	Latency	Small	-0.63	6.78	0.01	1.000	0.000
		Large	-16.63	10.23	2.64	.434	0.035
P3a	Amplitude	Small	0.13	0.33	0.16	.941	0.002
		Large	0.28	0.39	0.52	.941	0.007
	Latency	Small	19.58	10.09	3.77	.434	0.050
		Large	0.00	8.90	0.00	1.000	0.000

Table S2. Results of mixed-effects repeated measures examining the interaction between Group (Control, Sleep) and Session (Pre, Post) under the attend condition, separately for each Deviant type (Small, Large) and each dependent variable. Reported statistics include F-values with corresponding degrees of freedom (df_1, df_2), q = FDR-corrected p values (Benjamini–Hochberg), partial η^2 = effect size (small ≈ .01, medium ≈ .06, large ≈ .14). For each measure, parameter estimates (β) and standard errors (SE) are also presented.

ERP	Measure	Deviant	Estimate	SE	$F_{(1,76)}$	q	partial η^2
N2b	Amplitude	Small	0.31	0.32	0.93	.748	0.012
		Large	-0.09	0.22	0.17	.849	0.002
	Latency	Small	-4.67	8.73	0.29	.778	0.004
		Large	-17.20	7.41	5.38	.762	0.066
P3b	Amplitude	Small	-0.11	0.56	0.04	.778	0.001
		Large	0.39	0.41	0.90	.748	0.012
	Latency	Small	-8.05	11.25	0.51	.184	0.007
		Large	-7.73	8.65	0.80	.748	0.010

Table S3. Results of mixed-effects repeated measures examining the interaction between Group (Control, Sleep) and Session (Pre, Post) for behavioral data, separately for each Deviant type (Small, Large) and each dependent variable. Reported statistics include F-values with corresponding degrees of freedom (df_1, df_2), q = FDR-corrected p values (Benjamini–Hochberg), partial η^2 = effect size (small ≈ .01, medium ≈ .06, large ≈ .14). For each measure, parameter estimates (β) and standard errors (SE) are also presented.

Measure	Deviant	Estimate	SE	$F_{(1,64)}$	q	partial η^2
RT	Small	8.717	18.905	0.213	.775	0.003
	Large	8.389	15.392	0.297	.775	0.005
ACC	Small	-3.028	4.056	0.557	.775	0.009
	Large	-0.826	2.874	0.083	.775	0.001

Table S4. Mean and standard error for the amplitude (µV) and latency (ms) of the ERPs in the ignore condition separately for Deviant type, Group, and Session.

Condition	ERP	Measure	Deviant	Group	Session	Mean	SE
Ignore	MMN	Amplitude	Small	Control	Pre	-0.58	0.19
					Post	-0.62	0.24
				Sleep	Pre	-0.64	0.17
					Post	-0.54	0.22
			Large	Control	Pre	-1.12	0.22
					Post	-1.53	0.18
				Sleep	Pre	-0.93	0.19
					Post	-1.03	0.26
		Latency	Small	Control	Pre	239.37	6.20
					Post	230.11	6.66
				Sleep	Pre	239.79	7.12
					Post	231.16	7.24
			Large	Control	Pre	232.63	7.11
					Post	212.21	7.08
				Sleep	Pre	222.74	6.69
					Post	218.95	8.05
	P3a	Amplitude	Small	Control	Pre	0.04	0.22
					Post	0.59	0.22
				Sleep	Pre	-0.21	0.16
					Post	0.19	0.21
			Large	Control	Pre	-0.04	0.24
					Post	0.31	0.25
				Sleep	Pre	-0.11	0.22
					Post	-0.03	0.25
		Latency	Small	Control	Pre	288.84	6.09
					Post	305.05	5.86
				Sleep	Pre	286.95	5.75
					Post	283.58	6.50
			Large	Control	Pre	294.32	6.65
					Post	304.63	4.75
				Sleep	Pre	278.53	5.01
					Post	288.84	5.89

Table S5. Mean and standard deviation for the amplitude (μV) and latency (ms) of the ERPs in the attend condition separately for Deviant type, Group, and Session.

Condition	ERP	Measure	Deviant	Group	Session	Mean	SE
Attend	N2b	Amplitude	Small	Control	Pre	-1.10	0.18
					Post	-0.66	0.21
				Sleep	Pre	-0.76	0.26
					Post	-0.63	0.22
			Large	Control	Pre	-1.16	0.23
					Post	-1.23	0.27
				Sleep	Pre	-1.10	0.20
					Post	-1.08	0.21
		Latency	Small	Control	Pre	268.76	5.79
					Post	261.14	6.80
				Sleep	Pre	274.74	5.62
					Post	271.79	6.83
			Large	Control	Pre	252.76	4.46
					Post	240.19	4.61
				Sleep	Pre	256.42	5.72
					Post	261.05	7.12
	P3b	Amplitude	Small	Control	Pre	2.36	0.35
					Post	2.17	0.46
				Sleep	Pre	2.54	0.36
					Post	2.45	0.34
			Large	Control	Pre	2.88	0.34
					Post	3.00	0.43
				Sleep	Pre	2.91	0.33
					Post	2.64	0.41
		Latency	Small	Control	Pre	421.33	6.79
					Post	407.81	6.03
				Sleep	Pre	403.16	7.46
					Post	397.68	7.93
			Large	Control	Pre	415.62	6.90
					Post	399.05	6.80
				Sleep	Pre	398.95	7.19
					Post	390.11	6.15

Table S6. Mean and standard error for the behavioural accuracy (ACC, %) and reaction time (RT, ms) separately for the Deviant type, Group, and Session.

	Measure	Deviant	Group	Session	Mean	SE
Behaviour	ACC	Small	Control	Pre	86.78	3.54
				Post	92.06	2.60
			Sleep	Pre	92.75	1.74
				Post	95.00	1.39
		Large	Control	Pre	93.28	2.83
				Post	95.17	1.27
			Sleep	Pre	97.31	0.98
				Post	98.38	0.73
	RT	Small	Control	Pre	420.79	21.53
				Post	377.83	20.46
			Sleep	Pre	380.20	9.02
				Post	345.95	10.08
		Large	Control	Pre	387.03	19.39
				Post	356.23	17.74
			Sleep	Pre	344.35	8.66
				Post	321.93	8.59

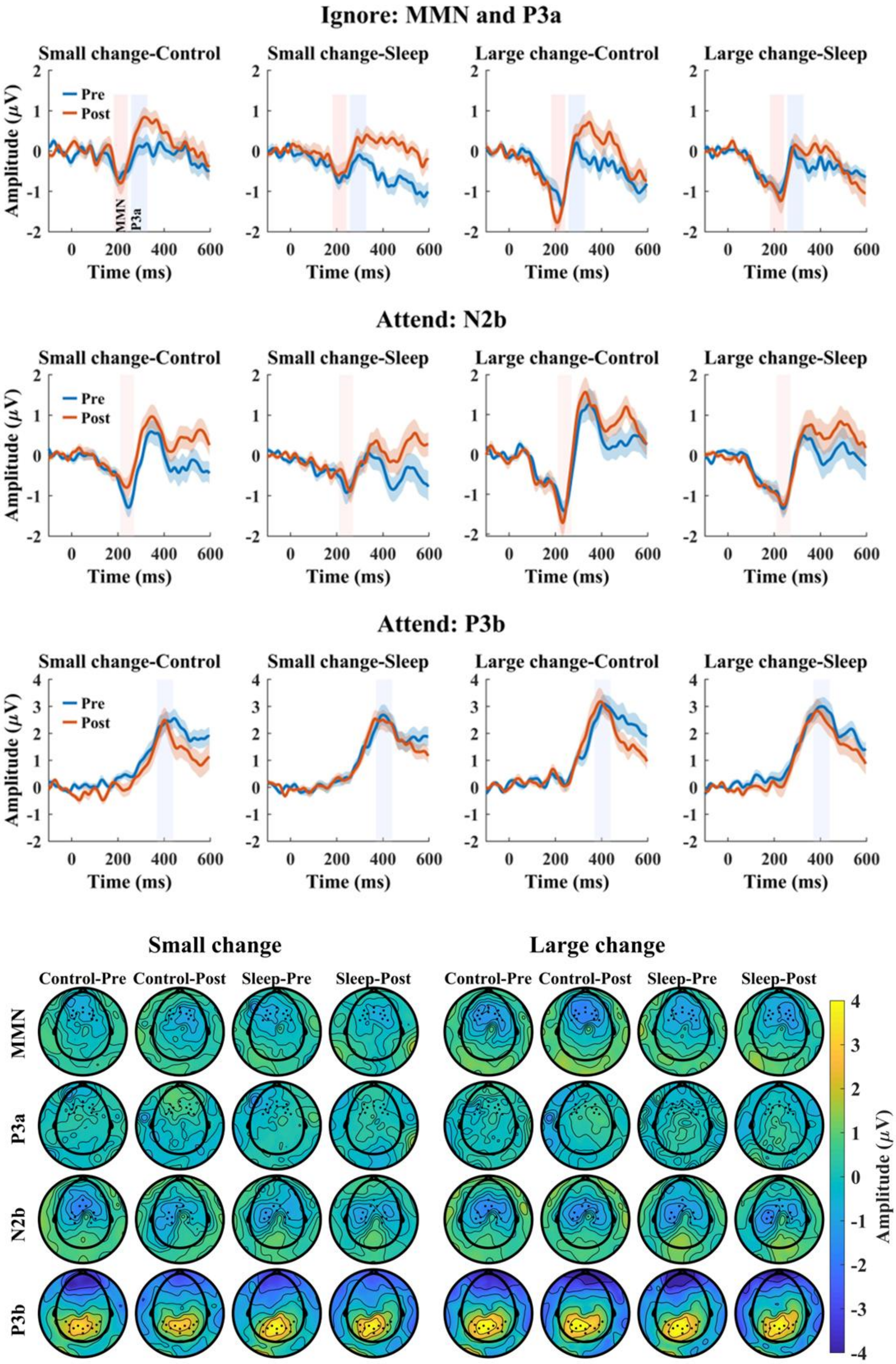

Figure S1. Grand-average differential responses and scalp topographies for the *Control* and *Sleep* groups under *Ignore* and *Attend* conditions, and for small and large changes.

Upper panels: ERP waveforms are shown for each group, session (Control-Pre, Sleep-Pre, Control-Post, Sleep-Post) and deviant condition (Small change, Large change) at representative

frontal and central electrode clusters. Shaded areas represent ±1 standard error (SE). Shaded bars indicate the time windows used for the amplitude analyses of the MMN (182 - 242 ms), N2b (210 - 270 ms), P3a (257 - 327 ms), and P3b (369 - 439 ms) components.
Lower panels: Scalp topographies averaged across the respective time windows are displayed separately for each ERP component and condition. Black dots indicate the electrode clusters used for calculating the mean amplitudes of the respective components.

Reference

Delorme A, Makeig S. 2004. EEGLAB: an open source toolbox for analysis of single-trial EEG dynamics including independent component analysis. *Journal of Neuroscience Methods.* 134:9–21. https://doi.org/10.1016/j.jneumeth.2003.10.009.

Maris E, Oostenveld R. 2007. Nonparametric statistical testing of EEG- and MEG-data. *Journal of Neuroscience Methods.* 164:177–190. https://doi.org/10.1016/j.jneumeth.2007.03.024.

Kurkela, J. L., Hämäläinen, J. A., Leppänen, P. H., Shu, H., & Astikainen, P. (2019). Passive exposure to speech sounds modifies change detection brain responses in adults. *NeuroImage*, *188*, 208-216. https://doi.org/10.1016/j.neuroimage.2018.12.010
